# Supplementary material for: Alcohol Sensor Based on Surface Plasmon Resonance of ZnO Nanoflowers/Au Structure
Source: Materials (Basel). 2021 Dec 27;15(1):189. doi: 10.3390/ma15010189 (PMC8745978; doi:10.3390/ma15010189)
Supplement: Supplementary file 1 [file materials-15-00189-s001.zip › materials-1502556-supplementary.pdf]

materials

Supporting Information

Alcohol Sensor Based on Surface Plasmon Resonance of ZnO Nanoflowers/Au Structure

Haowen Xu [1], Yutong Song [1], Panpan Zhu [1], Wanli Zhao [2], Tongyu Liu [2], Qi Wang [1,]* and Tianming Zhao [1,]*

[1] College of Sciences, Northeastern University, Shenyang 110819, China; xuhaowen@stumail.neu.edu.cn (H.X.); 2000172@stu.neu.edu.cn (Y.S.); zhupanpan2004@163.com (P.Z.);
[2] Science and Technology on Electro-Optical Information Security Control Laboratory, Tianjin 300308, China; wanlizhao@163.com(W.Z.); liu_tongyu@163.com(T.L.);
* Correspondence: wangqi@mail.neu.edu.cn (Q.W.); zhaotm@stumail.neu.edu.cn (T.Z.)

NUMBER OF PAGES: S2

NUMBER OF FIGURES: S2

NUMBER OF TABLES: S3

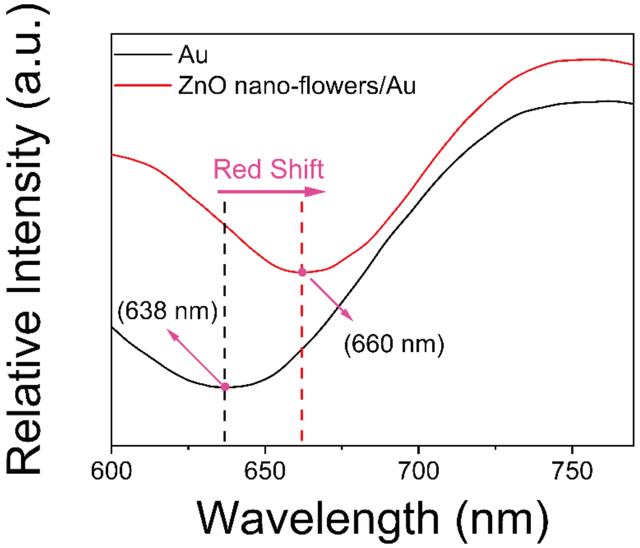

Figure S1. SPR signal of water (0 vol%) by Au film and ZnO nanoflowers/Au structure, respectively.

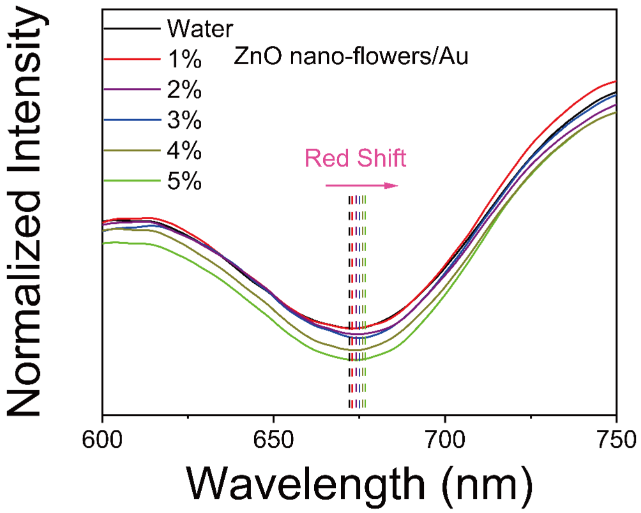

Figure S2. The LOD of the ZnO nanoflowers/Au structure.

Table S1. Comparison of measurement results between refraction and various concentrations of alcohol.

Concentrations of Alcohol (VOL%)	Reflective Index (RI)
0	1.332
5	1.335
15	1.3396
25	1.3466
35	1.3521
45	1.357
55	1.3605
65	1.3625
75	1.3662
85	1.3705
95	1.3721

Table S2. Comparison of measurement results between refraction and various concentrations of formaldehyde.

Concentrations of formaldehyde (VOL%)	Reflective Index (RI)
10	1.3440
15	1.3496
20	1.3553
25	1.3613
30	1.366

Table S3. Comparison of measurement results between refraction and various concentrations of formamide.

Concentrations of formamide (VOL%)	Reflective Index (RI)
10	1.3457
20	1.3581
30	1.370
40	1.3821
50	1.3937
